# Supplementary figures and images for: Site-specific targeting of a light activated dCas9-KillerRed fusion protein generates transient, localized regions of oxidative DNA damage
Source: PLoS One. 2020 Dec 17;15(12):e0237759. doi: 10.1371/journal.pone.0237759 (PMC7746297; doi:10.1371/journal.pone.0237759)

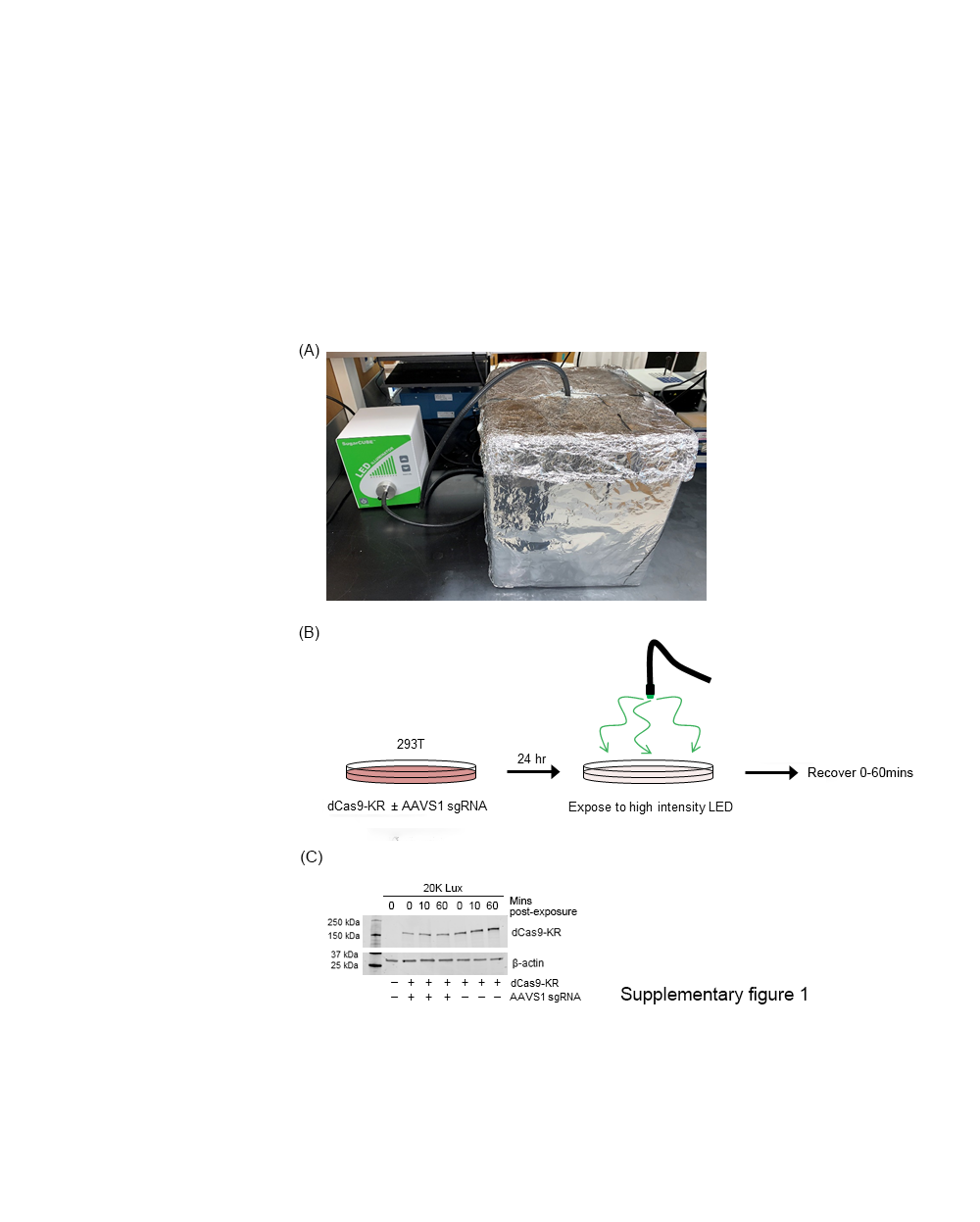

Supplement: S1 Fig — (A) Photograph of actual apparatus for light delivery. The SugarCUBE LED with a green bulb is attached via a liquid light guide to an aluminum foil protected Styrofoam box. Adherent cells in plates were placed inside the box with the lid closed and the LED was turned on for the times indicated in individual experiments. Light intensity was measured using a luxometer placed underneath the plate of cells. Intensity was measured in lux, the SI unit of illuminance which is a measure of the amount of light emitted per second per square meter. (B) Typical experimental schematic. 293T cells were transiently transfected with the dCas9-KR and/or the AAVS1 sgRNA. At 24hr post-transfection, cells were illuminated by exposure to the SugarCUBE LED and then placed back in the incubator at 37°C for recovery before cell collection. Illumination and recovery times varied by experiment. (C) 293T cells were transiently transfected with either vector (-), dCas9-KR or AAVS1 gRNA as indicated. 24 hrs later, cells were exposed to 20K Lux for the indicated times, followed by western blot analysis to detect dCas9-KR and b-actin (loading control). (TIF) [file pone.0237759.s001.tif]

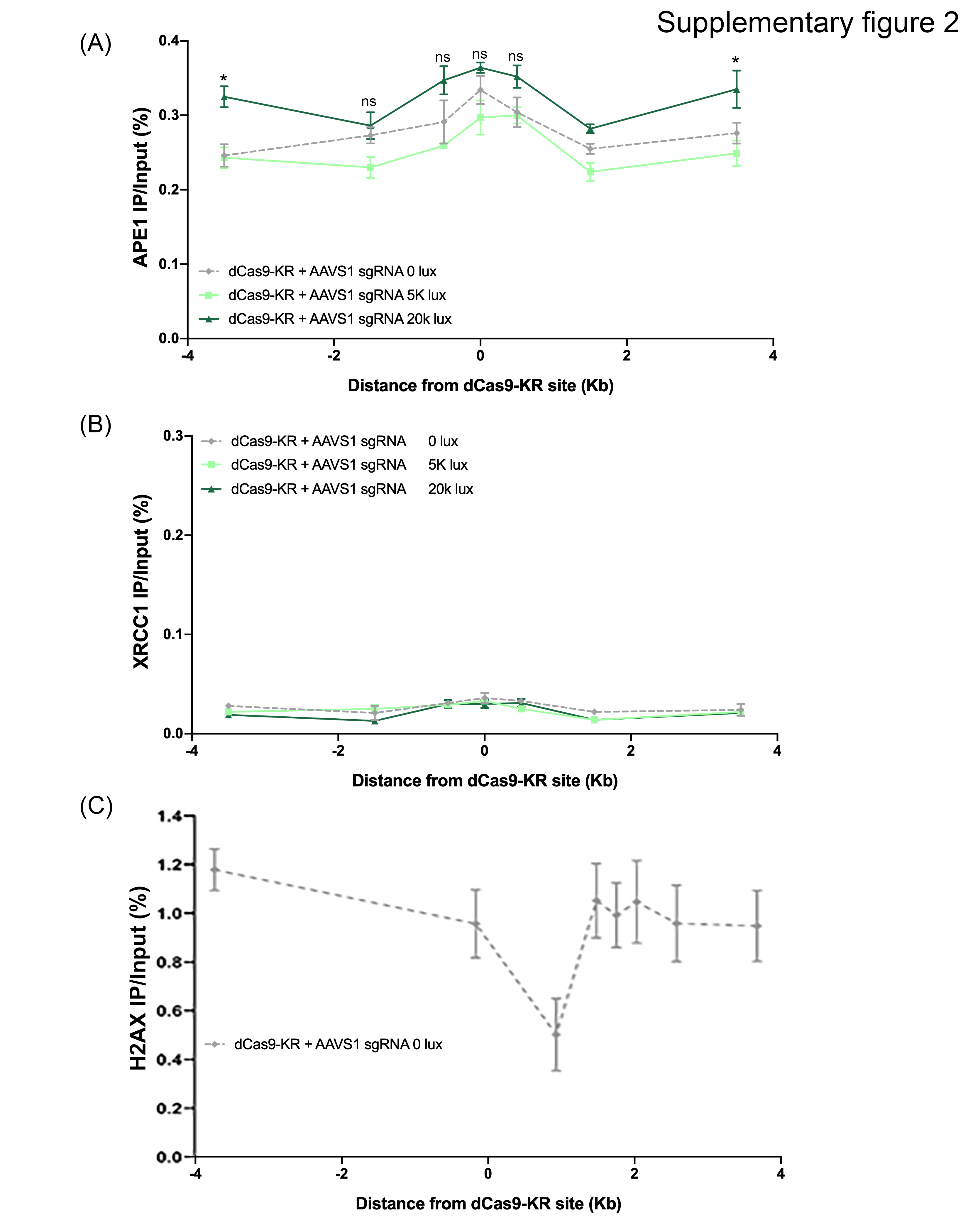

Supplement: S2 Fig — (A) ChIP for APE1 following transfection of dCas9-KR plus the indicated sgRNA. ChIP was carried out using APE1 antibody and primer pairs at the indicated distance from the dCas9-KR site (located at 0 base pairs). * = p < 0.05; ns = non-specific. (B) As in (A), but using XRCC1 antibody. (C) as in (A), but using H2AX antibody to monitor relative H2AX occupancy at the indicated positions. All methods described in materials and methods section. (TIF) [file pone.0237759.s002.tif]

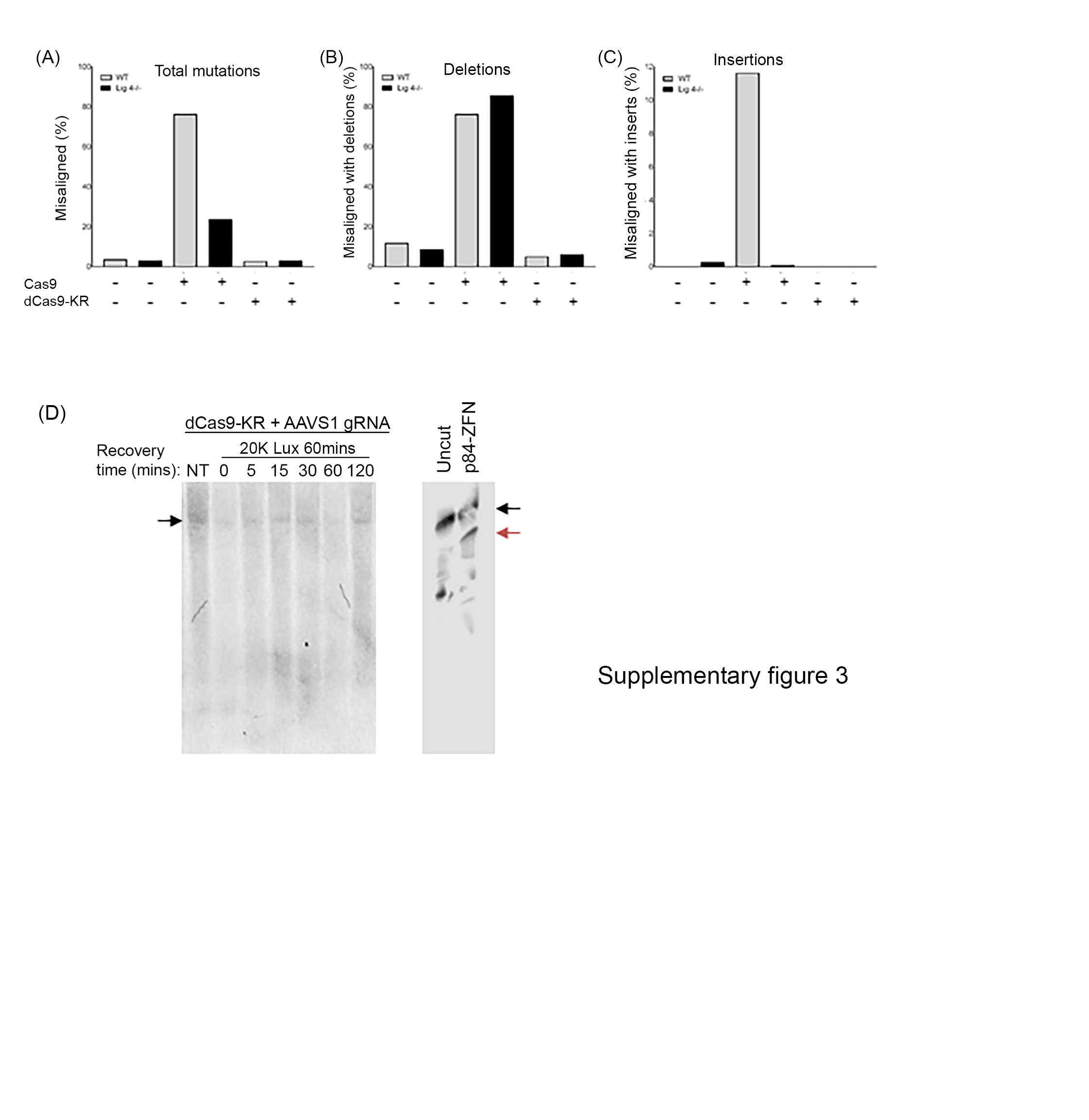

Supplement: S3 Fig — Wild type or Ligase IV-/- 293T cells were transfected with vector, nuclease proficient Cas9 plus gRNA or dCas9-KR. dCas9-KR cells were illuminated at 1hr at 20K lux. DNA was isolated 48 hr post-illumination and a 241 bp fragment surrounding the dCas9-KR was amplified and sequenced by NGS. (A) Total misalignments compared to the reference sequence (non-transfected cells); (B) deletions; (C) insertions; (D) DNA was isolated at the indicated time points post-illumination (0–120 minutes at 20K lux), followed by Southern blot to measure chromosome breakage at the AAVS1 site (left panel). The p84-ZFN nuclease was used as a positive control for DSB-induction (right panel, arrows). NT = not treated. The AAVS1 site was detected using a biotin-16-dUTP labeled amplicon spanning the AAVS1 site. (TIF) [file pone.0237759.s003.tif]

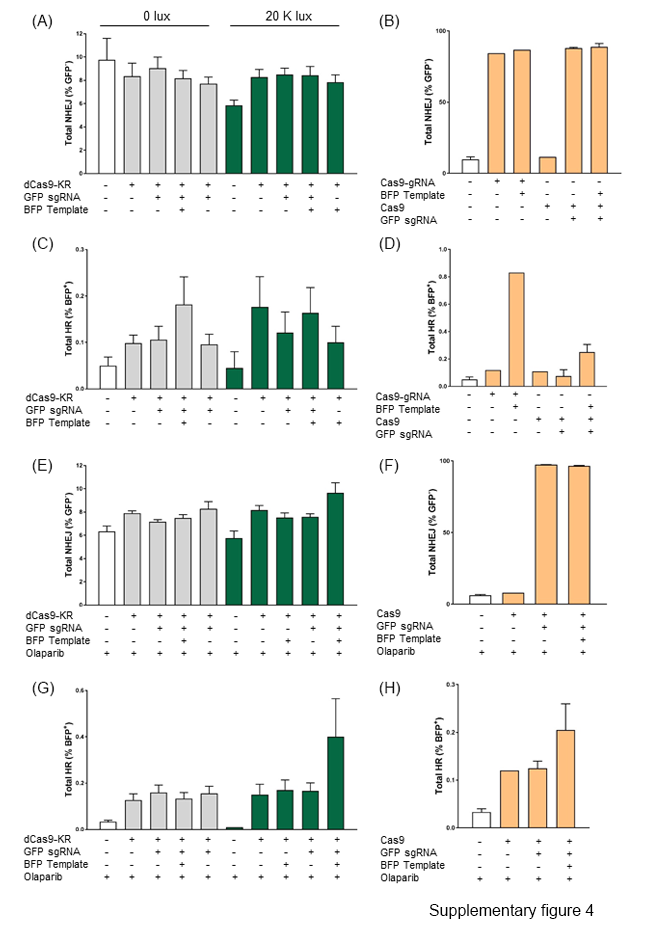

Supplement: S4 Fig — In this assay, cells contain a GFP array that can be targeted by the dCas-KR construct. Frameshift mutations after repair convert GFP+ to GFP−and this is used as a measure of NHEJ efficiency after DNA damage at a GFP array. When a BFP template is provided, repair can proceed via homologous recombination, resulting in gene conversion from GFP+ to BFP+. Inefficient DNA damage induction or infrequent mutational repair can result in GFP+BFP+ cells due to several copies of GFP being present in the target array. We have therefore scored any BFP+ cell as having undergone gene conversion/HR. The mean and error for at least three biological replicates are plotted. (A) Conversion from GFP+ to GFP−to measure NHEJ after dCas9-KR ROS-induced DSB repair in untreated (0 lux) or illuminated (30K lux, 1 hr) cells. The percent of GFP−cells are plotted to represent cells that underwent NHEJ. (B) Nuclease proficient Cas9 constructs were used as positive controls. Cas9 and the guide RNA were either in the same vector (Cas9-gRNA) or co-transfected as separate vectors (Cas9, GFP sgRNA). (C) Conversion to BFP+ to measure HR frequency after dCas9-KR ROS-induced DSB repair, performed as in (A). (D) Nuclease proficient Cas9 used as positive controls as in (B). (E-H) GFP to BFP conversion assay in the presence of Olaparib. Given that the dCas9-KR-induced DNA damage appeared to be quickly repaired (Figs 2E, 2F and 3E), we attempted to increase the frequency of DSBs by using olaparib, which traps PARP on the DNA, potentially limiting BER repair and causing DSBs (40, 41). Cells containing the dCas9-KR and GFP-targeted sgRNA were pre-treated in 25 uM Olaparib for two hours prior to illumination, and Olaparib was removed one hour post-illumination. For comparison, nuclease proficient Cas9 plus gRNA were also used. (E) dCas9-KR activation in Olaparib treated cells did not increase NHEJ (GFP–) rates, and therefore NHEJ is relatively unaffected or occurring at levels that are too low to be detected by [file pone.0237759.s004.tif]

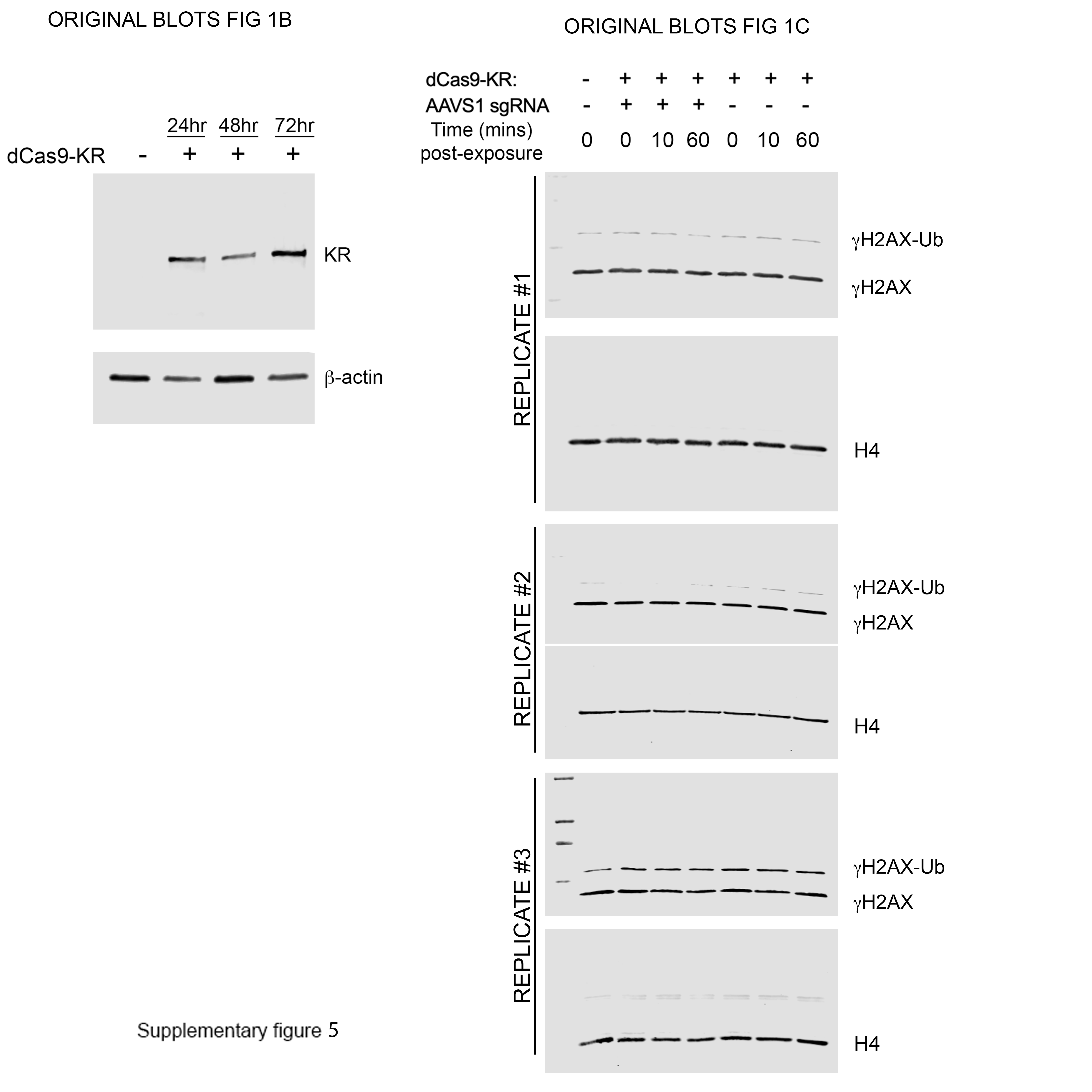

Supplement: S5 Fig — (TIF) [file pone.0237759.s005.tif]
