# Supplementary material for: Site-specific targeting of a light activated dCas9-KillerRed fusion protein generates transient, localized regions of oxidative DNA damage
Source: PLoS One. 2020 Dec 17;15(12):e0237759. doi: 10.1371/journal.pone.0237759 (PMC7746297; doi:10.1371/journal.pone.0237759)
Supplement: S1 File — (DOCX) [file pone.0237759.s006.docx]

| **Antibody** | **Catalog Number** | **Use** |
| --- | --- | --- |
| β-actin | Cell Signaling Technologies 4967L | Western blot |
| γH2AX | Abcam ab81299 | ChIP |
| γH2AX | Cell Signaling Technologies 2577S | Western blot |
| APE1 | Abcam ab194 | ChIP |
| BRCA1 | Abcam ab16781 | ChIP |
| H4ac | EMD Millipore 06-866 | ChIP |
| H2AX | Abcam ab11175 | ChIP |
| KillerRed | Evrogen AB961 | ChIP and Western blot |
| Ku70/80 | Neomarkers MS-286-P1 | ChIP |
| RPA | Millipore NA19L/Abcam ab2175 | ChIP |
| XRCC1 | Abcam ab1838 | ChIP |

| **Primer Name** | **Primer Sequence** | **Use** |
| --- | --- | --- |
| AAVS1 -10 kb Forward | AGGAGAGTATACGGGTTTGGCAC | ChIP qPCR amplification |
| AAVS1 -10 kb Reverse | AACTCCCCTATCCTACCCATCCA | ChIP qPCR amplification |
| AAVS1 -3.5 kb Forward | CCGAGATCACATCACTGCAC | ChIP qPCR amplification |
| AAVS1 -3.5 kb Reverse | GGAAAGAGGAGGGAAGAGGA | ChIP qPCR amplification |
| AAVS1 -1.5 kb Forward | GGGGCAGTCTGCTATTCATC | ChIP qPCR amplification |
| AAVS1 -1.5 kb Reverse | CGATGCACACTGGGAAGTC) | ChIP qPCR amplification |
| AAVS1 -0.5 kb Forward | TGGGTTCCCTTTTCCTTCTC | ChIP qPCR amplification |
| AAVS1 -0.5 kb Reverse | GTCCAGGCAAAGAAAGCAAG | ChIP qPCR amplification |
| AAVS1 0 kb Forward | CGGTTAATGTGGCTCTGGTT | ChIP qPCR amplification |
| AAVS1 0 kb Reverse | ACAGGAGGTGGGGGTTAGAC | ChIP qPCR amplification |
| AAVS1 +0.5 kb Forward | TTGGTTTCTTGCCTTGTTCC | ChIP qPCR amplification |
| AAVS1 +0.5 kb Reverse | GGCCTCTGATGGTGATTGTT | ChIP qPCR amplification, Southern probe amplification |
| AAVS1 +1.5 kb Forward | TGCCAATTAAATTTTTAGTTCTCTT | ChIP qPCR amplification |
| AAVS1 +1.5 kb Reverse | AACCTTCACCATTGCCATTC | ChIP qPCR amplification |
| AAVS1 +3.5 kb Forward | AGGGCTGGCCTCTAAAATCT | ChIP qPCR amplification |
| AAVS1 +3.5 kb Reverse | AAGGAACTCGTGGGGTTTTT | ChIP qPCR amplification |
| AAVS1 -80 bp Forward | GACCACCTTATATTCCCAGG | NGS fragment amplification, Southern probe amplification |
| AAVS1 +161 bp Reverse | GAGGTTCTGGCAAGGAGAGA | Southern probe amplification |
| BFP Forward | GCGACGTAAACGGCCACAAG | Amplification of BFP for donor template |
| BFP Reverse | GCCCTCGAACTTCACCTCG |  |

| **sgRNA name** | **sgRNA target sequence** | **Source** |
| --- | --- | --- |
| sgAAVS1 | GGGGCCACTAGGGACAGGAT | Addgene #41818 |
| sgeGFP | GGCTGAAGCACTGCACGCCGT | Genecopoeia |

dCas9-KR DNA sequence:

AAGCTGGCTAGCGCCGCCACC....ATGGACAAGAAGTACTCCATTGGGCTCGCTATCGGCACAAACAGCGTCGGCTGGGCCGTCATTACGGACGAGTACAAGGTGCCGAGCAAAAAATTCAAAGTTCTGGGCAATACCGATCGCCACAGCATAAAGAAGAACCTCATTGGCGCCCTCCTGTTCGACTCCGGGGAGACGGCCGAAGCCACGCGGCTCAAAAGAACAGCACGGCGCAGATATACCCGCAGAAAGAATCGGATCTGCTACCTGCAGGAGATCTTTAGTAATGAGATGGCTAAGGTGGATGACTCTTTCTTCCATAGGCTGGAGGAGTCCTTTTTGGTGGAGGAGGATAAAAAGCACGAGCGCCACCCAATCTTTGGCAATATCGTGGACGAGGTGGCGTACCATGAAAAGTACCCAACCATATATCATCTGAGGAAGAAGCTTGTAGACAGTACTGATAAGGCTGACTTGCGGTTGATCTATCTCGCGCTGGCGCATATGATCAAATTTCGGGGACACTTCCTCATCGAGGGGGACCTGAACCCAGACAACAGCGATGTCGACAAACTCTTTATCCAACTGGTTCAGACTTACAATCAGCTTTTCGAAGAGAACCCGATCAACGCATCCGGAGTTGACGCCAAAGCAATCCTGAGCGCTAGGCTGTCCAAATCCCGGCGGCTCGAAAACCTCATCGCACAGCTCCCTGGGGAGAAGAAGAACGGCCTGTTTGGTAATCTTATCGCCCTGTCACTCGGGCTGACCCCCAACTTTAAATCTAACTTCGACCTGGCCGAAGATGCCAAGCTTCAACTGAGCAAAGACACCTACGATGATGATCTCGACAATCTGCTGGCCCAGATCGGCGACCAGTACGCAGACCTTTTTTTGGCGGCAAAGAACCTGTCAGACGCCATTCTGCTGAGTGATATTCTGCGAGTGAACACGGAGATCACCAAAGCTCCGCTGAGCGCTAGTATGATCAAGCGCTATGATGAGCACCACCAAGACTTGACTTTGCTGAAGGCCCTTGTCAGACAGCAACTGCCTGAGAAGTACAAGGAAATTTTCTTCGATCAGTCTAAAAATGGCTACGCCGGATACATTGACGGCGGAGCAAGCCAGGAGGAATTTTACAAATTTATTAAGCCCATCTTGGAAAAAATGGACGGCACCGAGGAGCTGCTGGTAAAGCTTAACAGAGAAGATCTGTTGCGCAAACAGCGCACTTTCGACAATGGAAGCATCCCCCACCAGATTCACCTGGGCGAACTGCACGCTATCCTCAGGCGGCAAGAGGATTTCTACCCCTTTTTGAAAGATAACAGGGAAAAGATTGAGAAAATCCTCACATTTCGGATACCCTACTATGTAGGCCCCCTCGCCCGGGGAAATTCCAGATTCGCGTGGATGACTCGCAAATCAGAAGAGACCATCACTCCCTGGAACTTCGAGGAAGTCGTGGATAAGGGGGCCTCTGCCCAGTCCTTCATCGAAAGGATGACTAACTTTGATAAAAATCTGCCTAACGAAAAGGTGCTTCCTAAACACTCTCTGCTGTACGAGTACTTCACAGTTTATAACGAGCTCACCAAGGTCAAATACGTCACAGAAGGGATGAGAAAGCCAGCATTCCTGTCTGGAGAGCAGAAGAAAGCTATCGTGGACCTCCTCTTCAAGACGAACCGGAAAGTTACCGTGAAACAGCTCAAAGAAGACTATTTCAAAAAGATTGAATGTTTCGACTCTGTTGAAATCAGCGGAGTGGAGGATCGCTTCAACGCATCCCTGGGAACGTATCACGATCTCCTGAAAATCATTAAAGACAAGGACTTCCTGGACAATGAGGAGAACGAGGACATTCTTGAGGACATTGTCCTCACCCTTACGTTGTTTGAAGATAGGGAGATGATTGAAGAACGCTTGAAAACTTACGCTCATCTCTTCGACGACAAAGTCATGAAACAGCTCAAGAGGCGCCGATATACAGGATGGGGGCGGCTGTCAAGAAAACTGATCAATGGGATCCGAGACAAGCAGAGTGGAAAGACAATCCTGGATTTTCTTAAGTCCGATGGATTTGCCAACCGGAACTTCATGCAGTTGATCCATGATGACTCTCTCACCTTTAAGGAGGACATCCAGAAAGCACAAGTTTCTGGCCAGGGGGACAGTCTTCACGAGCACATCGCTAATCTTGCAGGTAGCCCAGCTATCAAAAAGGGAATACTGCAGACCGTTAAGGTCGTGGATGAACTCGTCAAAGTAATGGGAAGGCATAAGCCCGAGAATATCGTTATCGAGATGGCCCGAGAGAACCAAACTACCCAGAAGGGACAGAAGAACAGTAGGGAAAGGATGAAGAGGATTGAAGAGGGTATAAAAGAACTGGGGTCCCAAATCCTTAAGGAACACCCAGTTGAAAACACCCAGCTTCAGAATGAGAAGCTCTACCTGTACTACCTGCAGAACGGCAGGGACATGTACGTGGATCAGGAACTGGACATCAATCGGCTCTCCGACTACGACGTGGCTGCTATCGTGCCCCAGTCTTTTCTCAAAGATGATTCTATTGATAATAAAGTGTTGACAAGATCCGATAAAgcTAGAGGGAAGAGTGATAACGTCCCCTCAGAAGAAGTTGTCAAGAAAATGAAAAATTATTGGCGGCAGCTGCTGAACGCCAAACTGATCACACAACGGAAGTTCGATAATCTGACTAAGGCTGAACGAGGTGGCCTGTCTGAGTTGGATAAAGCCGGCTTCATCAAAAGGCAGCTTGTTGAGACACGCCAGATCACCAAGCACGTGGCCCAAATTCTCGATTCACGCATGAACACCAAGTACGATGAAAATGACAAACTGATTCGAGAGGTGAAAGTTATTACTCTGAAGTCTAAGCTGGTCTCAGATTTCAGAAAGGACTTTCAGTTTTATAAGGTGAGAGAGATCAACAATTACCACCATGCGCATGATGCCTACCTGAATGCAGTGGTAGGCACTGCACTTATCAAAAAATATCCCAAGCTTGAATCTGAATTTGTTTACGGAGACTATAAAGTGTACGATGTTAGGAAAATGATCGCAAAGTCTGAGCAGGAAATAGGCAAGGCCACCGCTAAGTACTTCTTTTACAGCAATATTATGAATTTTTTCAAGACCGAGATTACACTGGCCAATGGAGAGATTCGGAAGCGACCACTTATCGAAACAAACGGAGAAACAGGAGAAATCGTGTGGGACAAGGGTAGGGATTTCGCGACAGTCCGGAAGGTCCTGTCCATGCCGCAGGTGAACATCGTTAAAAAGACCGAAGTACAGACCGGAGGCTTCTCCAAGGAAAGTATCCTCCCGAAAAGGAACAGCGACAAGCTGATCGCACGCAAAAAAGATTGGGACCCCAAGAAATACGGCGGATTCGATTCTCCTACAGTCGCTTACAGTGTACTGGTTGTGGCCAAAGTGGAGAAAGGGAAGTCTAAAAAACTCAAAAGCGTCAAGGAACTGCTGGGCATCACAATCATGGAGCGATCAAGCTTCGAAAAAAACCCCATCGACTTTCTCGAGGCGAAAGGATATAAAGAGGTCAAAAAAGACCTCATCATTAAGCTTCCCAAGTACTCTCTCTTTGAGCTTGAAAACGGCCGGAAACGAATGCTCGCTAGTGCGGGCGAGCTGCAGAAAGGTAACGAGCTGGCACTGCCCTCTAAATACGTTAATTTCTTGTATCTGGCCAGCCACTATGAAAAGCTCAAAGGGTCTCCCGAAGATAATGAGCAGAAGCAGCTGTTCGTGGAACAACACAAACACTACCTTGATGAGATCATCGAGCAAATAAGCGAATTCTCCAAAAGAGTGATCCTCGCCGACGCTAACCTCGATAAGGTGCTTTCTGCTTACAATAAGCACAGGGATAAGCCCATCAGGGAGCAGGCAGAAAACATTATCCACTTGTTTACTCTGACCAACTTGGGCGCGCCTGCAGCCTTCAAGTACTTCGACACCACCATAGACAGAAAGCGGTACACCTCTACAAAGGAGGTCCTGGACGCCACACTGATTCATCAGTCAATTACGGGGCTCTATGAAACAAGAATCGACCTCTCTCAGCTCGGTGGAGACAGCAGGGCTGACCCCAAGAAGAAGAGGAAGGTGAAACCGGTCGCCACCATGGGTTCAGAGGGCGGCCCCGCCCTGTTCCAGAGCGACATGACCTTCAAAATCTTCATCGACGGCGAGGTGAACGGCCAGAAGTTCACCATCGTGGCCGACGGCAGCAGCAAGTTCCCCCACGGCGACTTCAACGTGCACGCCGTGTGCGAGACCGGCAAGCTGCCCATGAGCTGGAAGCCCATCTGCCACCTGATCCAGTACGGCGAGCCCTTCTTCGCCCGCTACCCCGACGGCATCAGCCATTTCGCCCAGGAGTGCTTCCCCGAGGGCCTGAGCATCGACCGCACCGTGCGCTTCGAGAACGACGGCACCATGACCAGCCACCACACCTACGAGCTGGACGACACCTGCGTGGTGAGCCGCATCACCGTGAACTGCGACGGCTTCCAGCCCGACGGCCCCATCATGCGCGACCAGCTGGTGGACATCCTGCCCAACGAGACCCACATGTTCCCCCACGGCCCCAACGCCGTGCGCCAGCTGGCCTTCATCGGCTTCACCACCGCCGACGGCGGCCTGATGATGGGCCACTTCGACAGCAAGATGACCTTCAACGGCAGCCGCGCCATCGAGATCCCCGGCCCACACTTCGTGACCATCATCACCAAGCAGATGAGGGACACCAGCGACAAGCGCGACCACGTGTGCCAGCGCGAGGTGGCCTACGCCCACAGCGTGCCCCGCATCACCAGCGCCATCGGTAGCGACGAGGATTAAGAATTC

Key:

Cas9m4

KillerRed

Linker

AgeI site

EcoRI site

NheI site

DNA 2.0 default 5’ end

dCas9-KR amino acid sequence:

MDKKYSIGLAIGTNSVGWAVITDEYKVPSKKFKVLGNTDRHSIKKNLIGALLFDSGETAEATRLKRTARRRYTRRKNRICYLQEIFSNEMAKVDDSFFHRLEESFLVEEDKKHERHPIFGNIVDEVAYHEKYPTIYHLRKKLVDSTDKADLRLIYLALAHMIKFRGHFLIEGDLNPDNSDVDKLFIQLVQTYNQLFEENPINASGVDAKAILSARLSKSRRLENLIAQLPGEKKNGLFGNLIALSLGLTPNFKSNFDLAEDAKLQLSKDTYDDDLDNLLAQIGDQYADLFLAAKNLSDAILLSDILRVNTEITKAPLSASMIKRYDEHHQDLTLLKALVRQQLPEKYKEIFFDQSKNGYAGYIDGGASQEEFYKFIKPILEKMDGTEELLVKLNREDLLRKQRTFDNGSIPHQIHLGELHAILRRQEDFYPFLKDNREKIEKILTFRIPYYVGPLARGNSRFAWMTRKSEETITPWNFEEVVDKGASAQSFIERMTNFDKNLPNEKVLPKHSLLYEYFTVYNELTKVKYVTEGMRKPAFLSGEQKKAIVDLLFKTNRKVTVKQLKEDYFKKIECFDSVEISGVEDRFNASLGTYHDLLKIIKDKDFLDNEENEDILEDIVLTLTLFEDREMIEERLKTYAHLFDDKVMKQLKRRRYTGWGRLSRKLINGIRDKQSGKTILDFLKSDGFANRNFMQLIHDDSLTFKEDIQKAQVSGQGDSLHEHIANLAGSPAIKKGILQTVKVVDELVKVMGRHKPENIVIEMARENQTTQKGQKNSRERMKRIEEGIKELGSQILKEHPVENTQLQNEKLYLYYLQNGRDMYVDQELDINRLSDYDVAAIVPQSFLKDDSIDNKVLTRSDKARGKSDNVPSEEVVKKMKNYWRQLLNAKLITQRKFDNLTKAERGGLSELDKAGFIKRQLVETRQITKHVAQILDSRMNTKYDENDKLIREVKVITLKSKLVSDFRKDFQFYKVREINNYHHAHDAYLNAVVGTALIKKYPKLESEFVYGDYKVYDVRKMIAKSEQEIGKATAKYFFYSNIMNFFKTEITLANGEIRKRPLIETNGETGEIVWDKGRDFATVRKVLSMPQVNIVKKTEVQTGGFSKESILPKRNSDKLIARKKDWDPKKYGGFDSPTVAYSVLVVAKVEKGKSKKLKSVKELLGITIMERSSFEKNPIDFLEAKGYKEVKKDLIIKLPKYSLFELENGRKRMLASAGELQKGNELALPSKYVNFLYLASHYEKLKGSPEDNEQKQLFVEQHKHYLDEIIEQISEFSKRVILADANLDKVLSAYNKHRDKPIREQAENIIHLFTLTNLGAPAAFKYFDTTIDRKRYTSTKEVLDATLIHQSITGLYETRIDLSQLGGDSRADPKKKRKVKPVATMGSEGGPALFQSDMTFKIFIDGEVNGQKFTIVADGSSKFPHGDFNVHAVCETGKLPMSWKPICHLIQYGEPFFARYPDGISHFAQECFPEGLSIDRTVRFENDGTMTSHHTYELDDTCVVSRITVNCDGFQPDGPIMRDQLVDILPNETHMFPHGPNAVRQLAFIGFTTADGGLMMGHFDSKMTFNGSRAIEIPGPHFVTIITKQMRDTSDKRDHVCQREVAYAHSVPRITSAIGSDED

BFP g-Block

AGCTGGACGGCGACGTAAACGGCCACAAGTTCAGCGTGTCCGGCGAGGGAGAGGGCGATGCCACCTACGGCAAACTGACCCTGAAGTTCATCTGCACCACTGGCAAGCTGCCCGTGCCCTGGCCAACCCTCGTGACCACCCTGAGCCACGGGGTGCAGTGCTTCAGCCGCTACCCAGACCACATGAAGCAGCACGACTTTTTCAAGTCCGCCATGCCCGAAGGCTATGTCCAGGAGCGCACCATCTTCTTTAAGGACGACGGCAACTACAAGACCCGCGCCGAGGTGAAGTTCGAGGGCG
