# Supplementary material for: Site-specific targeting of a light activated dCas9-KillerRed fusion protein generates transient, localized regions of oxidative DNA damage
Source: PLoS One. 2020 Dec 17;15(12):e0237759. doi: 10.1371/journal.pone.0237759 (PMC7746297; doi:10.1371/journal.pone.0237759)
Supplement: Attachment — Submitted filename: Response to reviewers.docx [file pone.0237759.s011.docx]

**Response to reviewers**.

**Reviewer 1**.

Reviewer 1 requested additional clarification of the conclusion that dCas9-KR generates site-specific DNA damage, rather than general (locus specific) DNA damage due to ROS production throughout the nucleus. To address this, as requested, we repeated the γH2AX experiments in figure 1, using 3 biological replicates, with image quantification. This data is now included in figure 1, with the replicate blots used to generate the data presented in supplementary figure 5. This clearly shows that dCas9-KR activation does not lead to detectable increases in global γH2AX. Further, the ChIP data (figure 1) shows that γH2AX is only increased at the AAVS1 locus when both the targeting gRNA and light exposure are used, indicating that this damage requires docking of the dCas9-KR onto the chromatin to produce detectable damage. Although looking for specific γH2AX foci at the AAVS1 site would be a useful approach, in our initial characterization of the dCas9-KR system, we were unable to reproducibly detect γH2AX foci directly at the AAVS1 site. We believe this is due to the low γH2AX signal generated by dCas9-KR and the limited spreading of γH2AX from the site. However, ChIP, which is a more sensitive approach (figure 1), clearly shows the presence of increased γH2AX at the dCas9-KR locus. Taken together, we believe that this supports our original conclusion that dCas9-KR creates site-specific rather than global DNA damage.

(i) The original figure 2C was, in fact, incorrectly labelled. Since the changes in APE1 are small and were shown in more detail in the original supplementary figure 2, we have removed figure 2C and limit our discussion of this to the APE1 data set originally presented (supplementary figure 2A).
(ii) The difference in template integrity in figure 4A is not statistically significant. This was not made clear in the original submission, and we have provided statistical analysis in figure 4A and the figure 4 legend, as well as altered the text (lines 192-194) to reflect this.
(iii) The legend and figure 2B have been corrected to reflect that the exposure was 0, 10 and 60mins.
(iv) We have corrected line 219 (line 240 in the corrected version) and added “error-prone”.
(v) We have checked the labeling of figure 4E and figure 4F and these are now correctly annotated.

**Reviewer 2**.

We appreciate reviewer 2’s enthusiasm for the paper and thank them for their strong endorsement.

We have made the following changes to the statistical analysis, as requested:

- We have added statistical analysis to Figure 2, Figure 3E (now figure 3D in revised version) and Figures 4D and 4E, as requested. In figure 2C, for clarity, statistical significance is noted in the figure legend rather than on the figure.
- Figure 2C in the original submission has now been removed, as noted above (point (i)), with the data now presented in supplementary figure 2.
- A description of the statistics in Figure 4D, 4E, 4F and 4G has been added to the figure legend.
